# Humidity-Sensing Performance of TiO_2_/RGO and α-Fe_2_O_3_/RGO Composites

**DOI:** 10.3390/s25030691

**Published:** 2025-01-24

**Authors:** Wanghui Zou, Chenhui Wu, Wei Zhao

**Affiliations:** School of Physical and Electronic Sciences, Changsha University of Science and Technology, Changsha 410114, China; 23111022054@stu.csust.edu.cn (C.W.); zw1009220094@163.com (W.Z.)

**Keywords:** humidity sensor, nanocomposite material, room temperature, TiO_2_/RGO, α-Fe_2_O_3_/RGO

## Abstract

This study investigates the humidity-sensing properties of two semiconductor metal oxide (SMO)-reduced graphene oxide (RGO) nanocomposites: TiO_2_/RGO and α-Fe_2_O_3_/RGO, at room temperature. Both nanocomposites are synthesized via hydrothermal methods and coated onto printed circuit board (PCB) interdigital electrodes to construct humidity sensors. The surface morphology and crystallographic structure of the materials are characterized using field emission scanning electron microscopy (FESEM) and X-ray diffraction (XRD). The sensors are tested across a humidity range of 11%RH to 97%RH, and the impedance is measured over a frequency range of 1 Hz to 1 MHz. The results show that both TiO_2_/RGO and α-Fe_2_O_3_/RGO exhibit favorable humidity-sensing performance at room temperature. The sensitivity and humidity hysteresis of TiO_2_/RGO are 12.2 MΩ/%RH and 3.811%RH, respectively, while those of α-Fe_2_O_3_/RGO are 0.826 MΩ/%RH and 8.229%RH. The response and recovery times of TiO_2_/RGO are 72 s and 99 s, respectively, while those of α-Fe_2_O_3_/RGO are 48 s and 54 s. Both sensors demonstrate good repeatability and stability. These findings suggest that SMO/RGO nanocomposites are promising materials for the development of low-cost, high-sensitivity, and stable humidity sensors.

## 1. Introduction

Humidity sensors are of great significance in the fields of medicine, biochemistry, industrial production, animal husbandry and agriculture, scientific research, and food processing [1,2,3]. As the basis and core of humidity sensors, the moisture-sensitive materials directly or indirectly absorb the water molecules in the detected environment, which changes the chemical or physical properties of the materials, thereby achieving the purpose of humidity detection. Although several novel humidity sensors have been developed in recent years, such as Quartz Crystal Microbalance (QCM) sensors, Optical Fiber Humidity Sensors, Capacitive Micro-machined Ultrasonic Transducer (CMUT) sensors, and Film Bulk Acoustic Resonator (FBAR) sensors [4], that demonstrate significant potential for achieving higher sensitivity, accuracy, and stability, the classic electrical humidity sensors based on the resistance, capacitance, or impedance characteristics of sensitive materials remain the most widely used. These sensors continue to attract considerable attention due to their simple structures, low cost, small size, and well-established application solutions. Furthermore, the development of new sensitive materials, such as nanocomposite materials, has led to continuous performance improvements, further enhancing their practical value.

The sensitive materials used for electrical humidity sensors mainly include electrolytes [5], organic polymers [6], metal oxides [7], ceramics [8], and mixed materials [9]. Among them, organic polymers are the most traditional materials, and semiconductor metal oxide (SMO) materials are widely studied due to their great variety, simple preparation process, and good performance. SMO materials respond to many factors in the environment. Humidity, temperature, pressure, illumination, the concentration of oxidizing or reducing gases, and so forth can change the condition of the surface of the materials [10] and result in changes in the electrical properties of the materials. The good electrical response of SMO materials to environmental humidity has promoted the development and application of SMO humidity sensors. However, the humidity-sensing performance of single-SMO material is usually limited, which limits the application of SMO humidity sensors. On the other side, composite materials based on multiple common materials or a mixture of common materials with novel low-dimensional materials show great potential for providing superior sensing performance [9].

As a new type of carbon nanomaterials, reduced graphene oxide (RGO) has high electron mobility, high electrical conductivity, and other superior characteristics. Meanwhile, according to previous research, it is found that RGO material also has some humidity sensitivity [11]. Due to the unique nano-structure and physical and chemical properties of RGO, there is a potential that the combination of RGO and SMO materials has a humidity-sensing performance improvement over pure SMO materials. In [12], Yan H et al. synthesized SnO_2_/RGO nanocomposite materials using a one-step hydrothermal method. Experiment results reveal that the addition of RGO effectively improves the performance of the sensor. The SnO_2_/RGO nanocomposite materials with different RGO doping ratios show different humidity-sensing properties. For instance, the response and the recovery time of the 1 wt% SnO_2_/RGO are 180 s and 8 s, respectively, which is approximately twice those of 2 wt% SnO_2_/RGO. This further illustrates the positive impact of adding RGO. In [13], Morsy M et al. synthesized RGO/Fe_2_O_3_ nanocomposite materials using a precipitation technique and lyophilization process. The response and the recovery time of the RGO/Fe_2_O_3_ sensor are 63 s and 48 s, respectively. Compared with Fe_2_O_3_, the introduction of RGO produces more active sites, such as vacancies and defects, which promote the adsorption of water molecules and, therefore, improve the sensitivity of the sensor. In addition to metal oxide materials, hydrophobic polymers can also form sensing composite materials with RGO. In [14], Fanyu Y et al. also studied the humidity-sensing properties of RGO/SnO_2_ composites prepared using a hydrothermal method. It was stated that, compared with SnO_2_, the incorporation of RGO makes the RGO/SnO_2_ composites have a larger specific surface area and more hydrophilic functional groups and oxygen vacancy defects, which makes the sensor have better linearity and smaller humidity hysteresis. In [15], a flexible humidity sensor was fabricated based on polyvinylidene fluoride PVDF/RGO composite materials using a solvent casting method. Compared with PVDF, the sensor demonstrates higher sensitivity, faster response/recovery times (21/26 s), better stability, and smaller hysteresis within a relative humidity (RH) range of 11–97%. As the concentration of RGO increases, the sensitivity of the sensor increases accordingly and reaches 98.99% at 0.3 vol%.

In this paper, we focus mainly on SMO/RGO composite materials, specifically, TiO_2_/RGO and α-Fe_2_O_3_/RGO. The humidity-sensing properties of the two composite materials at room temperature are examined, including sensitivity, hysteresis, response/recovery time, impedance characteristics, repeatability, and stability. Also, the differences between the two materials are analyzed and explained.

## 2. Experiment

### 2.1. Raw Materials and Instrument

Chemicals used in experiments were all of analytical grade (AR). Graphene oxide (GO) aqueous solution (5 mg/mL) was purchased from Tanfeng Graphene Technology Co., Ltd. in Suzhou, China. Tetrabutyl titanate aqueous solution (1 g/mL) and ferric chloride hexahydrate were both obtained from Wuxi Yatai United Chemical Co., Ltd. in Wuxi, China. Hydrochloric acid was purchased from Anzexin Technology Co., Ltd. in Shenzhen, China. Anhydrous ethanol was purchased from Xirong Chemical Co., Ltd. in Shantou, China.

The Box furnace (KSL-1200X-A2, Jingke, Hangzhou, China) was used for material calcination. The physical phase of the samples was characterized by X-ray diffraction (XRD, Rigaku Ultima IV, Rigaku Corporation, Tokyo, Japan). The morphology and microstructure of the samples were studied by scanning electron microscope (SEM, apro2, Thermo Fisher, Tokyo, Japan). Impedance spectroscopy tests were conducted using an electrochemical workstation (CS350H, Coster, Wuhan, China).

### 2.2. Synthesis of Materials

Synthesis of TiO_2_/RGO: Firstly, 4 mL of GO aqueous solution and 10 mL of anhydrous ethanol were added to 20 mL of deionized water, and the mixed solution was magnetically stirred for 20 min to ensure uniformity, resulting in solution A. Secondly, 10 mL of tertbutyl titanate aqueous solution was dispersed in 20 mL of anhydrous ethanol, and hydrochloric acid was added to adjust the pH value to approximately 3. After magnetic stirring for 30 min, solution B was obtained. Thirdly, solution A and solution B were mixed together and magnetically stirred again and then transferred to a Teflon-lined hydrothermal reactor. Then, the whole set was placed into a blast drying oven for a hydrothermal reaction at 160 °C for 12 h. Finally, After the precipitate cooled to room temperature, it was washed repeatedly with deionized water and anhydrous ethanol to remove impurities and then dried at 80 °C for 2 h. The washed and dried precipitate was then ground into powder, and the TiO_2_/RGO composite was obtained. The RGO accounts for 1% of the total mass in the TiO_2_/RGO composite.

Synthesis of α-Fe_2_O_3_/RGO: Firstly, 1.08 g of ferric chloride hexahydrate was dissolved into 30 mL of deionized water and magnetically stirred for 20 min, and therefore, solution A was obtained. Secondly, 10 mL of GO aqueous solution was added to 30 mL of deionized water. After 10 min magnetic stirring, solution B was obtained. Thirdly, solutions A and solution B were mixed together and transferred to a Teflon-lined hydrothermal reactor, which was then placed into a blast drying oven for a hydrothermal reaction at 180 °C for 10 h. Finally, after the precipitate cooled to room temperature, it was processed in the same way as mentioned above, and the α-Fe_2_O_3_/RGO composite was obtained. The RGO accounts for 4.5% of the total mass in the α-Fe_2_O_3_/RGO composite.

### 2.3. Fabrication of Sensors

**Figure 1:**
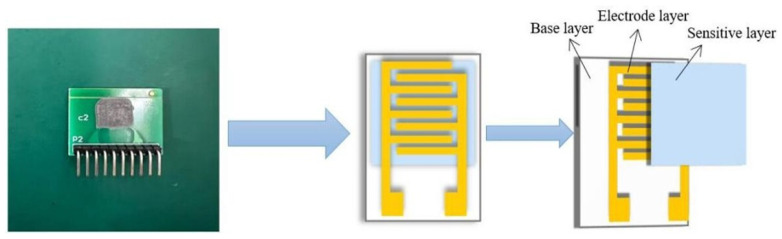
Schematic and structure of the humidity sensor.

The regular printed circuit board (PCB) was used to fabricate the sensor substrate with Cu-Sn interdigital electrodes, which is depicted in Figure 1. The electrode length was designed to be 7.62 mm, and both the width and gap of the interdigitated fingers were designed to be 0.254 mm. The sensor fabrication process is as follows: Firstly, an appropriate amount of the prepared composite materials was placed in an agate mortar, and a small amount of anhydrous ethanol was added to disperse the materials into a slurry. Secondly, the mixed slurry was brushed onto the PCB substrate to cover the electrodes. The PCB substrate was then placed into an electric blast drying oven at 60 °C for 2 h to remove ethanol molecules and possible water molecules. As a result, the humidity sensor was obtained. Finally, the newly made sensor was placed on a heating stage at 70 °C for 8 h for aging to enhance stability.

### 2.4. Experiment System

**Figure 2:**
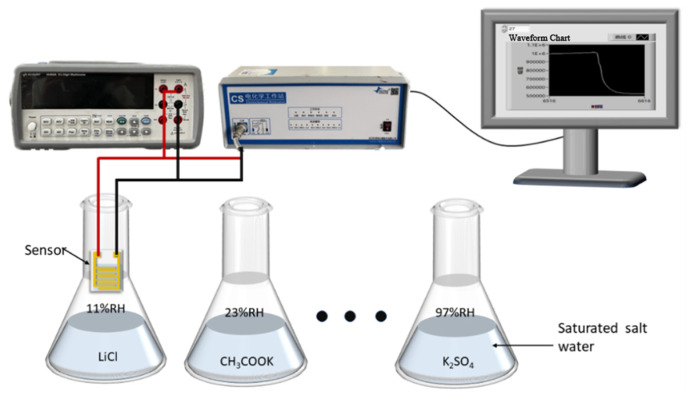
Experimental setup for humidity sensor testing.

Figure 2 shows the humidity test system, which consists of a set of saturated salt solutions, a digital multimeter (Keysight 34450A, Keysight, Santa Rosa, CA, USA), an electrochemical workstation, and a computer. According to previous studies, the saturated salt solutions stored in sealed glass vessels can provide a constant and stable relative humidity environment [16,17,18]. We selected 8 saturated salt solutions to form eight different relative humidity points in the range of 11%RH to 95%RH, as listed in Table 1, to construct a complete test setup. The fabricated humidity sensor was connected to the digital multimeter. When the humidity sensor was placed into various glass vessels, the digital multimeter measured and displayed the resistance change of the sensors. The computer was also connected to the digital multimeter via the serial port and communicated with it. We also built a testing program on the computer that obtained, recorded, and stored the data from the multimeter in real-time.

Through further analysis of the stored data, the performance parameters of the humidity sensor including sensitivity, response/recovery time, stability characteristics, and so on can be derived. The electrochemical workstation was used to measure the complex impedance and frequency response of the sensors. A professional software tool was used to conduct parameter fitting for the equivalent circuits of the sensors, which further aids in analyzing and verifying the sensing mechanism of the humidity sensor.

## 3. Humidity-Sensing Mechanism

The response of the humidity sensor is closely related to the adsorption and desorption state of water molecules on the surface of the sensing material. Generally, the adsorption of water molecules can be divided into two processes: physical adsorption and chemical adsorption. When the humidity sensor is exposed to a humid environment, the hydrogen atoms in the water molecules collide on the material surface, causing ionization of the water molecules to form a hydroxyl ion (OH^−^) and a proton (H^+^), as described by Equation (1). The hydroxyl ion is adsorbed by the metal cation on the surface of the material, and the proton reacts with the adjacent oxygen ion (O^2−^) to form a new hydroxyl ion, as described by Equation (2). The new hydroxyl ion will also be adsorbed by the metal cations. Therefore, the ionization of water molecules produces two hydroxyl ions and adsorbs on the composite materials, helping to form a chemical adsorption layer. On top of the chemical adsorption layer, subsequent water molecules are physically adsorbed through double hydrogen bonding and form the first physical adsorption layer. As the humidity further increases, more physical adsorption occurs, and multiple water layers are formed through single hydrogen bonding. In fact, the subsequent physical adsorption layers formed through single hydrogen bonding are not strong, and the water molecules are relatively easy to dissociate, which promotes the formation of H_3_O^+^ ions as described by Equation (3). H_3_O^+^ acts as the proton source and facilitates conduction in the form of proton hopping under external electric excitation. This process is known as the Grotthuss mechanism [19].

(1) $$H_{2}O\to H^{+}+{OH}^{-}$$

(2) $$H^{+}+O^{2-}\to {OH}^{-}$$

(3) $$H_{2}O+H^{+}\to {H_{3}O}^{+}$$

**Figure 3:**
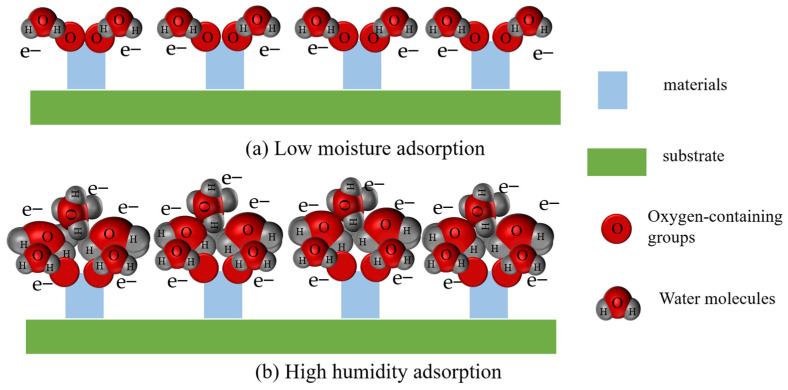
Humidity-sensing mechanism at (**a**) low and (**b**) high humidity levels.

As shown in Figure 3, at low relative humidity, a continuous water molecule layer cannot form, making it difficult for protons to transfer, and therefore, the sensor exhibits high impedance. In the case of high relative humidity, enough water molecules are adsorbed onto the surface of the material to form continuous water layers, and a large number of H_3_O^+^ ions are also generated. According to the Grotthuss mechanism, H_3_O^+^ ions are rapidly transferred and transported in the continuous water molecule layers ($H_{2}O+H_{3}O^{+}\to H_{3}O^{+}+H_{2}O$), leading to a significant drop in impedance and a large response from the sensor.

It is recognized that reduced graphene oxide exhibits a layered structure, with numerous hydrophilic functional groups on its surface. This unique structure, along with the hydrophilic functional groups, provides abundant adsorption sites for water molecules. The incorporation of RGO is believed to increase the specific surface area of the composite material, thereby enhancing the number of active sites available on its surface. When the composite material is exposed to a humid environment, a greater number of water molecules are adsorbed onto the surface of the metal oxide/RGO composite. These water molecules ionize to form hydroxide (OH^−^) ions, which are subsequently adsorbed by the metal cations on the surface of the composite or by the polar groups in the RGO. Protons (H^+^) then react with the oxygen ions (O^2−^) on the composite material’s surface, generating additional hydroxide ions (OH^−^). As a result, the incorporation of RGO into the composite material is likely to improve its humidity-sensitive properties.

## 4. Results and Discussions

### 4.1. Material Characterization

**Figure 4:**
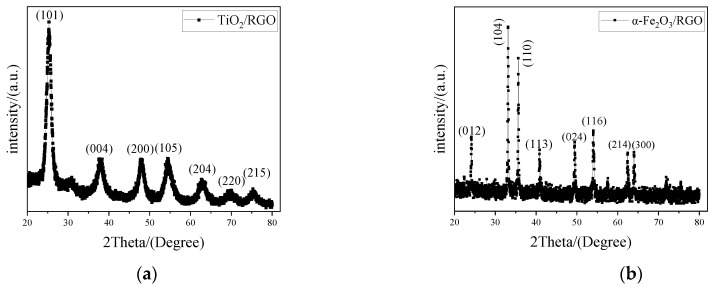
X-ray diffraction patterns of (**a**) TiO_2_/RGO and (**b**) α-Fe_2_O_3_/RGO.

The X-ray diffraction (XRD) patterns of TiO_2_/RGO and α-Fe_2_O_3_/RGO are depicted in Figure 4. In Figure 4a, there are diffraction peaks at 25.1°, 37.5°, 47.6°, 53.7°, 62.4°, 68.5°, and 74.7°, which clearly reflect the characteristic peaks of anatase TiO_2_ and can be indexed to (101), (004), (200), (105), (204), (220), and (215) crystal planes of anatase TiO_2_ [20]. In Figure 4b, the characteristic peaks appear at 24.1°, 33.2°, 35.6°, 40.9°, 49.5°, 54.1°, 62.4°, and 64.0°. The position and intensity of the diffraction peaks correspond to (012), (104), (110), (113), (024), (116), (214), and (330) crystal planes of α-Fe_2_O_3_ [21]. According to the literature, for RGO, there are diffraction peaks at 24.7° and 43.5°, which correspond to (002) and (101) crystal planes [22]. Because the content of RGO is relatively low, the characteristic peak of RGO at 24.7° is easily overshadowed by the characteristic peak of anatase TiO_2_ at 25.1°. Therefore, the XRD peaks of RGO disappear in TiO_2_/RGO composites. Similarly, the XRD peaks of RGO are also absent in α-Fe_2_O_3_/RGO composites due to the characteristic peak of α-Fe_2_O_3_ at 24.1°.

**Figure 5:**
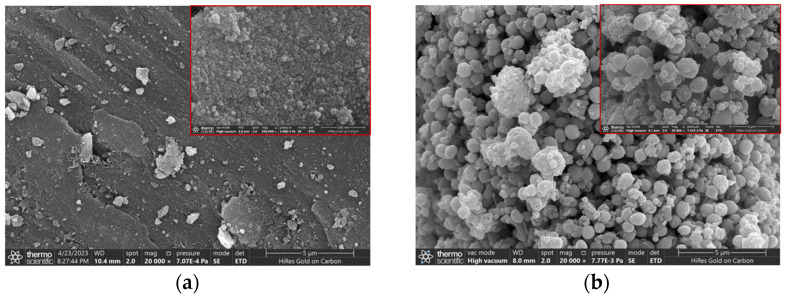
SEM image of (**a**) TiO_2_/RGO and (**b**) α-Fe_2_O_3_/RGO. An inset with a larger magnification is shown in the upper right corner. The magnification of the main images is 20,000×, while the magnifications of the inset images in (**a**,**b**) are 200,000× and 50,000×, respectively.

The scanning electron microscopy (SEM) of TiO_2/_RGO and α-Fe_2_O_3_/RGO is depicted in Figure 5. As seen in the image, the material exhibits a flaky, wrinkled structure with numerous gaps and an uneven surface. This suggests that the GO aqueous solution produced graphene oxide after a one-step hydrothermal reaction. According to the literature, RGO produced by a one-step hydrothermal reaction of GO aqueous solution typically exhibits an uneven surface and a flaky wrinkle structure with many voids [23]. Figure 5 shows the scanning electron microscopy (SEM) of the two composites with insets in a greater magnification. From Figure 5a, wrinkled flake structures can also be observed from the image, which indicates the presence of RGO in the material. As seen in the inset, the tiny spherical TiO_2_ nanoparticles are evenly dispersed on the RGO flakes, indicating that the TiO_2_/RGO composite material was generated after the hydrothermal reaction. From Figure 5b, the spherical α-Fe_2_O_3_ nanoparticles with a relatively large size are loosely piled. As can be found from the inset, RGO flakes are embedded between the α-Fe_2_O_3_ nanoparticles, indicating the formation of α-Fe_2_O_3_/RGO composites by a hydrothermal reaction.

### 4.2. Sensitivity and Hysteresis

**Figure 6:**
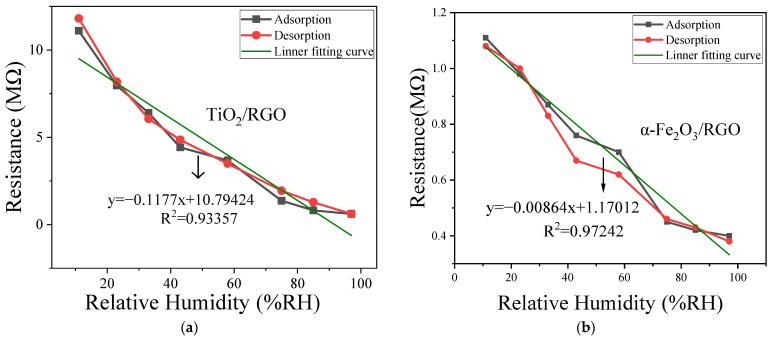
Hysteresis curves of (**a**) TiO_2_/RGO and (**b**) α-Fe_2_O_3_/RGO.

During the experiments, the room temperature was maintained at approximately 25 °C to eliminate the impact of temperature on the test results. Figure 6 shows the dynamic response of TiO_2_/RGO and α-Fe_2_O_3_/RGO. The two curves represent a hysteresis cycle, which includes absorption (the relative humidity changes gradually from 11%RH to 97%RH with a time interval of 10 min) and desorption (the relative humidity changes gradually from 97%RH to 11%RH with a time interval of 10 min) processes. Resistive humidity sensors typically show approximately linear response characteristics, so the usual data processing method is used to make a linear fitting on the response curves, followed by the calculation of sensitivity, fitting degree *R*^2^, and other parameters based on the measured data and fitting parameters [12,24]. At a certain initial humidity (set at 11%RH in this work), the resistance of the sensor is measured; then, the humidity is changed (set at 97%RH in this work), and the resistance is measured again. The sensitivity is generally defined as the ratio of the change in resistance (Δ*R*) to the change in humidity (Δ*RH*) as follows:

(4) $$S=\frac{∆R}{∆RH}\times 100\%,$$

Based on the linear fitting of the response curves, the slope is always taken as the overall sensitivity. The fitting degree *R*^2^ represents the linearity of the response curves. The closer it is to 1, the more linear the sensor response is [25]. The humidity hysteresis *H* is calculated with the sensitivity *S* and is defined as follows:

(5) $$H=\frac{R_{diff}}{S}\times 100\%,$$

where *R_diff_* is the maximum resistance difference between the absorption and the desorption curve.

From Figure 6, it can be seen that both composite materials exhibit a certain degree of linearity. For TiO_2_/RGO, the fitting degree *R*^2^ is 0.934, and the sensitivity at 97%RH is about 12.2 MΩ/%RH. The largest resistance difference *R_diff_* occurs at 75%RH, and the humidity hysteresis *H* is 3.811%RH. For α-Fe_2_O_3_/RGO, the fitting degree *R*^2^ is 0.972, and the sensitivity at 97%RH is about 0.826 MΩ/%RH. The maximum resistance difference appears at 43%RH, and the humidity hysteresis is 8.229%RH. Comparing these two materials, TiO_2_/RGO has better sensitivity and lower humidity hysteresis, while α-Fe_2_O_3_/RGO has better linearity.

Generally, humidity hysteresis always occurs, especially at room temperature, meaning there is always a certain degree of difference between the absorption and the desorption step. During the absorption step, water molecules are absorbed chemically or physically on the surface of the humidity-sensitive materials. However, during the desorption step, the previously absorbed water molecules cannot be quickly released from the surface of the material, which means that material resistance changes cannot follow the environmental humidity changes, and this leads to humidity hysteresis.

### 4.3. Response/Recovery Time

Response/recovery time is usually defined as the time required for the resistance change to reach 90% of the total change under a certain humidity step, which accurately reflects the speed of sensor output responding to humidity changes. In our experiments, the humidity sensor was first placed into the 11%RH vessel and allowed to stabilize. Then, the sensor was quickly transferred to the 97%RH vessel and allowed to stabilize again. The time from the start of the transfer to the time when the resistance change reaches 90% of the total change is obtained as the response time. Secondly, the sensor was quickly transferred back to the 11%RH vessel. Again, the time from the start of the transfer to the point where the resistance change reaches 90% of the total change is obtained as the recovery time. The response time relates to the absorption process, while the recovery time relates to the desorption process.

**Figure 7:**
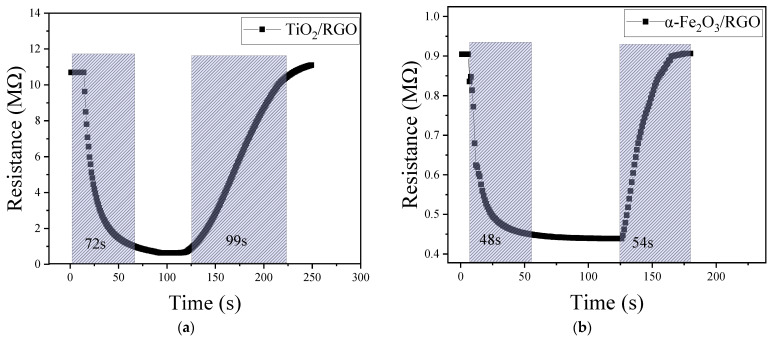
Response and recovery transients of (**a**) TiO_2_/RGO and (**b**) α-Fe_2_O_3_/RGO.

Figure 7 shows the response/recovery curves of the two composite materials. From the figures, the response and the recovery time of TiO_2_/RGO are approximately 72 s and 99 s, respectively, while those of α-Fe_2_O_3_/RGO are 48 s and 54 s, respectively. Comparing these two composite materials, α-Fe_2_O_3_/RGO shows a better response and recovery performance. Recalling the SEM images of α-Fe_2_O_3_/RGO and TiO_2_/RGO in Figure 5, loosely packed spherical α-Fe_2_O_3_ nanoparticles can be seen, and it seems that α-Fe_2_O_3_/RGO has a larger specific surface area compared with TiO_2_/RGO, which may help explain the advantages of α-Fe_2_O_3_/RGO in terms of the response/recovery time.

Table 2 lists and compares the response/recovery time, sensitivity, and sensing type of various humidity sensors in the literature and in this paper. All sensors operate at room temperature. It should be noted that there are multiple definitions of sensitivity in the literature. The sensitivities listed in Table 2 are estimated using the data from the literature and calculated according to Equation (2), primarily for the purpose of facilitating a comparison. It can be seen that the performance of the SMO/RGO composite materials is generally better than that of the mono-SMO materials or SMO composite materials. The main reason likely lies in the fact that the addition of RGO can promote the production of a larger specific surface area and more surface hydrophilic functional groups on the surface of the material, thereby improving the response/recovery performance of the humidity sensor. The SnO_2_/RGO composite materials described in [12,14] have a very short recovery time, whereas the two composite materials presented in this paper, especially α-Fe_2_O_3_/RGO, have relatively balanced and good response/recovery properties. According to [21], RGO can form a p-n heterojunction with TiO_2_ and α-Fe_2_O_3_, which may have a positive impact on the performance improvement of composite materials.

### 4.4. Complex Impedance and Frequency

**Figure 8:**
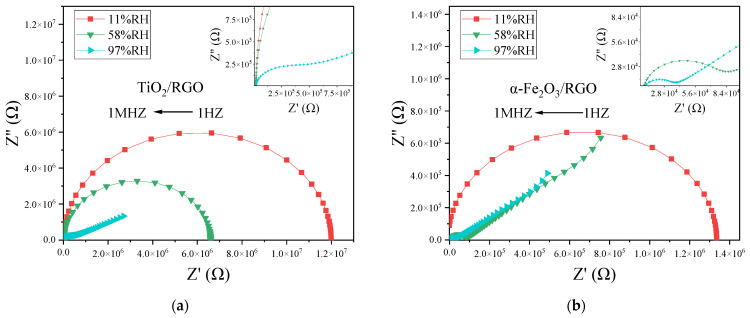
Complex impedance plot of (**a**) TiO_2_/RGO and (**b**) α-Fe_2_O_3_/RGO. The scanning frequency ranges from 1 Hz to 1 MHz.

To further explore the in-depth properties of materials, alternating current (AC) impedance measurements across a frequency range are highly useful. Electrochemical impedance spectroscopy (EIS) and corresponding equivalent circuits are essential tools for analyzing and studying the properties of sensitive materials and the sensing mechanisms of sensors. Figure 8 presents the Nyquist complex impedance diagrams of the TiO_2_/RGO sensor and the α-Fe_2_O_3_/RGO sensor under different relative humidity conditions (11%RH, 58%RH, and 97%RH). The scanning frequency ranges from 1 Hz to 1 MHz. At low humidity levels, the complex impedance primarily reflects the intrinsic impedance characteristics of the material films. The impedance of the TiO_2_/RGO film is much higher than that of the α-Fe_2_O_3_/RGO film. As the humidity increases, the complex impedance of the sensors further illustrates the interaction between the sensitive materials and water molecules.

**Figure 9:**
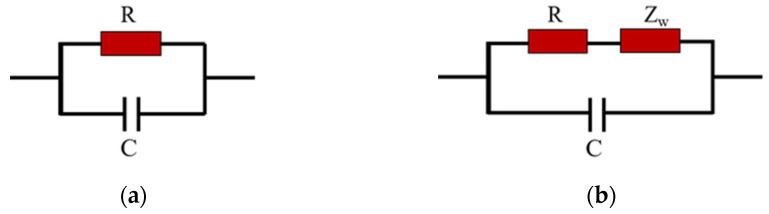
Typical equivalent circuit at (**a**) low and (**b**) high humidity conditions.

For TiO_2_/RGO, at a low relative humidity of 11%RH, only a small number of water molecules are chemically adsorbed on the surface of the material. High energy is required for H^+^ ions to transfer between adjacent hydroxyl groups via the hopping mechanism. Under low humidity conditions, the increase in humidity has a limited effect on the sensor’s impedance, which changes only slightly and remains high. As shown in Figure 8a, the complex impedance curve exhibits a shape close to an ideal semicircle. Typically, the equivalent circuit corresponding to this semicircle consists of a parallel connection of a resistance R and a capacitance C, as shown in Figure 9a. The diameter of the semicircle reflects the magnitude of the resistance R. When the humidity increases to a moderate level of 58%RH, more water molecules are adsorbed chemically or physically onto the surface of the material, gradually forming a continuous water layer. Proton hopping becomes more likely, resulting in a gradual decrease in impedance as humidity increases, and the semicircle’s radius decreases accordingly.

At a high humidity level of 97%RH, water molecules form a continuous, multi-layered water film on the surface of the material through physical adsorption. A large number of H_3_O^+^ ions transfer via the Grotthuss chain reaction, causing a significant reduction in impedance. More importantly, a “tail” appears at the low-frequency part of the semicircle. This tail is attributed to the Warburg impedance *Z_W_*, which arises from the ion diffusion process at the interface between the sensing film and the electrode and exhibits frequency-dependent characteristics [30]. Because of the Warburg impedance, the equivalent circuit is shown in Figure 9b.

The characteristics of α-Fe_2_O_3_/RGO differ somewhat from those of TiO_2_/RGO. As shown in Figure 8b, at a low humidity level of 11%RH, the complex impedance plot of α-Fe_2_O_3_/RGO also displays a nearly ideal semicircle, which is similar to that of TiO_2_/RGO. Thus, its equivalent circuit should also consist of a parallel combination of resistance R and capacitance C. However, when the humidity increases to a moderate level of 58%RH, the plot deviates from an ideal semicircle, and a tail emerges, differing from the behavior observed for TiO_2_/RGO. This is likely due to the loose structure of the α-Fe_2_O_3_/RGO material and its large specific surface area, which facilitates the rapid adsorption of water molecules into gaps, leading to the generation of a Warburg impedance.

Examining the change in the semicircle radius with humidity reveals that TiO_2_/RGO exhibits better linearity, while α-Fe_2_O_3_/RGO shows “radius jumps”. This discrepancy may also be related to the surface morphology of the materials. The surface of TiO_2_/RGO is relatively flat, allowing water molecules to distribute evenly and interact with the material in a way that produces a relatively linear effect. In contrast, the surface of α-Fe_2_O_3_/RGO is loose and irregular, with more voids, making it prone to generating non-linear effects.

Table 3 presents the fitting parameters for the equivalent circuit of TiO_2_/RGO and α-Fe_2_O_3_/RGO at different relative humidity levels. R and C represent the resistance and capacitance, respectively, in the equivalent circuit. The Warburg impedance *Z_ω_* can be calculated using the following equation [31]:

(6) $$Z_{\omega }=\frac{WoR\cdot ctnh({\left(j\omega WoT\right)}^{WoP})}{{\left(j\omega WoT\right)}^{WoP}}$$

where *ω* denotes the angular frequency, and *WoR*, *WoT*, and *WoP* are parameters of the Warburg impedance model. *WoR* represents the diffusion impedance at very low frequencies, *WoT* represents the diffusion time constant, and *WoP* reflects the effect of frequency on diffusion impedance. It can be observed that the *WoT* of TiO_2_/RGO is smaller than that of α-Fe_2_O_3_/RGO, indicating a faster ion diffusion process within the surface water layer of TiO_2_/RGO. On the other hand, the *WoP* of α-Fe_2_O_3_/RGO is greater than that of TiO_2_/RGO, suggesting that the diffusion impedance of α-Fe_2_O_3_/RGO is more sensitive to frequency. TiO_2_/RGO is a material with a relatively flat surface, which enables the formation of shorter diffusion pathways, resulting in a faster diffusion process at the interface between the TiO_2_/RGO material and the electrode. In contrast, ions in the α-Fe_2_O_3_/RGO material experience longer diffusion paths, making the diffusion process more susceptible to the changes in frequency.

**Figure 10:**
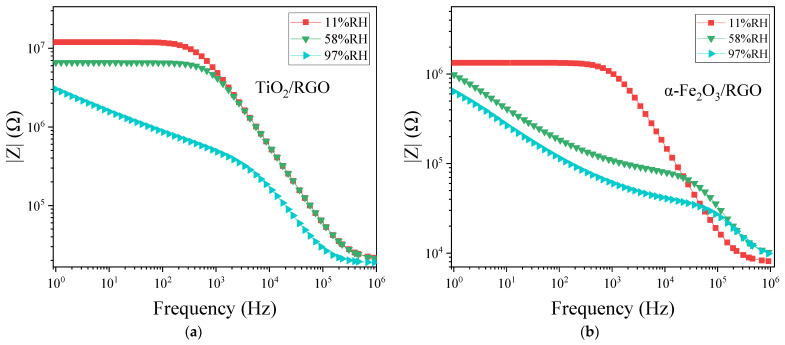
|Z| versus frequency (log(*f*)) of (**a**) TiO_2_/RGO and (**b**) α-Fe_2_O_3_/RGO under various RH.

Figure 10 illustrates the relationship between the impedance (|Z|) and frequency (log(*f*)) of the two sensors in the humidities of 11%RH, 58%RH, and 97%RH. Under low humidity conditions (such as 11%RH), |Z| changes slowly at low frequencies (around 1–10^3^ Hz), indicating an approximate DC resistive behavior in the material, while at high frequencies (around 10^3^–10^5^ Hz), the curves decrease linearly with frequency, exhibiting a certain degree of *f*^-−1^ behavior, indicating that the capacitance plays a dominant role. This is the typical impedance characteristic of the parallel combination of resistance and capacitance. Under high humidity conditions (such as 97%RH), the low-frequency portion of the curve is noticeably elevated, reflecting the influence of the Warburg impedance, as described earlier. Warburg impedance primarily affects the low-frequency characteristics and is approximately linearly related to frequency (log(*f*)).

### 4.5. Repeatability and Stability

**Figure 11:**
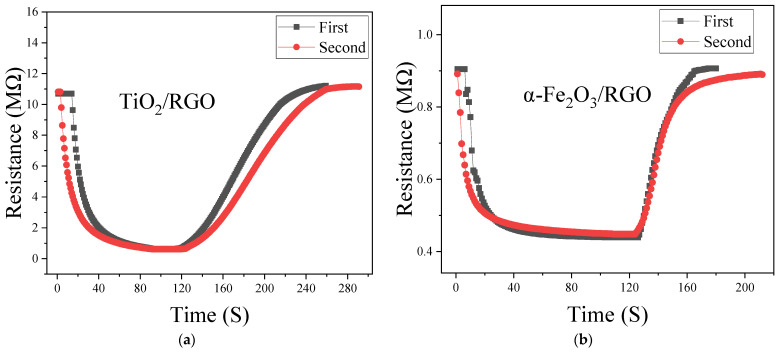
Repeatability test of (**a**) TiO_2_/RGO and (**b**) α-Fe_2_O_3_/RGO.

Repeatability and stability are essential properties of sensors. The test method for evaluating repeatability in this study is as follows: a response/recovery test is conducted, repeated after 15 days, and the differences between the two measured curves are compared. Figure 11 presents the repeatability test results for the two materials. Although there are some differences between the curves, it is evident that the sensor’s response and recovery processes are roughly consistent, indicating good repeatability. The discrepancies between the curves may arise from the following factors: first, the first test was performed immediately after the sensor was made, and the second test was conducted after the sensor had been placed in the environment for 15 days. In other words, the first test was based on a newly fabricated device, while the sensor may have undergone some changes after 15 days of exposure to the environment, making the results of the second test likely different; second, the experiments rely primarily on manual operations, and the procedure and environmental conditions may not be identical between the two tests.

**Figure 12:**
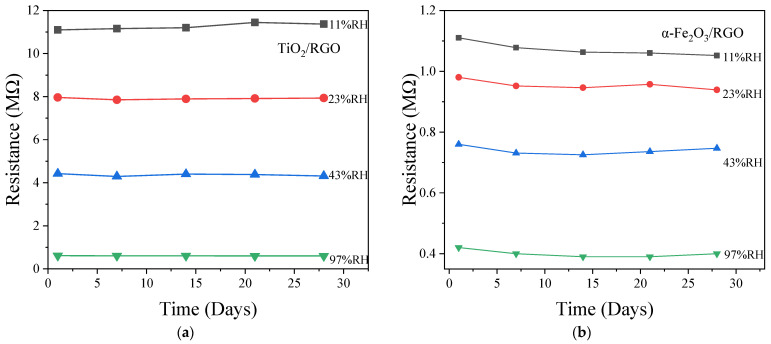
Stability curves of (**a**) TiO_2_/RGO and (**b**) α-Fe_2_O_3_/RGO in different humidities.

In addition, the sensors were subjected to a stability test by placing them in four different humidity environments (11%RH, 23%RH, 43%RH, and 97%RH) for approximately one month. The test results are shown in Figure 12. For the TiO_2_/RGO sensor, under humidity levels of 11%RH, 23%RH, 43%RH, and 97%RH, the maximum resistance variations were 0.24 MΩ, 0.11 MΩ, 0.13 MΩ, and 0.005 MΩ, respectively. For the α-Fe_2_O_3_/RGO sensor, the corresponding maximum resistance variations were 0.032 MΩ, 0.028 MΩ, 0.029 MΩ, and 0.02 MΩ, respectively. In percentage terms, the maximum resistance variations for both the TiO_2_/RGO and α-Fe_2_O_3_/RGO sensors were approximately 1% at 11%RH. Based on the test data, the TiO_2_/RGO sensor demonstrates relatively better stability. However, both sensors exhibit good overall stability performance.

## 5. Conclusions

This paper investigates the humidity-sensing properties of TiO_2_/RGO and α-Fe_2_O_3_/RGO nanocomposite materials at room temperature. The results show that both materials exhibit good humidity-sensing performance. The sensitivity and humidity hysteresis of TiO_2_/RGO were 12.2 MΩ/%RH and 3.811%RH, respectively, while those of α-Fe_2_O_3_/RGO were 0.826 MΩ/%RH and 8.229%RH, respectively. The response/recovery time of TiO_2_/RGO was 72/99 s, while that of α-Fe_2_O_3_/RGO was 48/54 s. Combined with other related literature, this study further confirms that the nanocomposites incorporating RGO will probably provide better moisture-sensitive performance than single material. Since the TiO_2_/RGO nanocomposite material exhibits a relatively large resistance, the sensor resistance changes greatly in a humid environment, which corresponds to a large absolute sensitivity. However, the relative sensitivity (responsivity) of the two materials is similar, indicating that the two materials all have good sensitivity. The α-Fe_2_O_3_/RGO has relatively better linearity from moderate to high humidity. Since α-Fe_2_O_3_/RGO has a relatively loose surface morphology, α-Fe_2_O_3_/RGO has a smaller response and recovery time, but it may also contribute to hysteresis. In cyclic testing, both materials showed good repeatability. In a one-month stability test under four humidity conditions of 11%RH, 23%RH, 43%RH, and 97%RH, both materials showed small fluctuation, indicating good stability. This study suggests that SMO-RGO composite materials, such as TiO_2_/RGO and α-Fe_2_O_3_/RGO, hold significant promise as candidate materials for developing high-sensitivity and high-stability humidity sensors, particularly suited for environmental monitoring in residential and commercial applications where cost-effectiveness is a critical consideration.

## Figures and Tables

**Table 1:** Relative humidity of saturated salt solution (25 °C).

Saturated Salt Solution	Relative Humidity (100%)
LiCl	11
CH_3_COOK	23
MgCl_2_	33
K_2_CO_3_	43
NaBr	58
NaCl	75
KCl	85
K_2_SO_4_	97

**Table 2:** Comparison of humidity-sensing performance.

Materials	Response (s)	Recovery (s)	Sensitivity	Sensing Type	Ref.
ZnO/TiO_2_	774.9	19.7	2.04 × 10^3^ pF/%RH	capacitance	[26]
RGO/SnO_2_	80	4	2.36 × 10^3^ pF/%RH	capacitance	[12]
α-Fe_2_O_3_	60	140	0.16 MΩ/%RH	impedance	[27]
ZrO_2_/TiO_2_	54	124	0.84 MΩ/%RH	impedance	[28]
RGO/Fe_2_O_3_	63	48	20.35 MΩ/%RH	impedance	[13]
SnO_2_/RGO	45	8	13.10 MΩ/%RH	impedance	[14]
PANI/α-Fe_2_O_3_	70	90	0.22 KΩ/%RH	resistance	[29]
α-Fe_2_O_3_/RGO	48	54	0.83 MΩ/%RH	resistance	This work
RGO/TiO_2_	72	99	12.20 MΩ/%RH	resistance	This work

**Table 3:** Fitting values of each element of the equivalent circuit at various humidity.

Humidity	R(Ω)	C(pF)	W_O_R(Ω)	WoT	W_O_P
11%RH (TiO_2_/RGO)	11.97 M	28.26	-	-	-
58%RH (TiO_2_/RGO)	6.573 M	28.04	-	-	-
97%RH (TiO_2_/RGO)	498.370 K	91.33	2.6257 M	0.17308	0.17417
11%RH (α-Fe_2_O_3_/RGO)	1.3432 M	98.67	-	-	-
58%RH (α-Fe_2_O_3_/RGO)	72.411 K	52.29	2.0592 M	0.83674	0.43813
97%RH (α-Fe_2_O_3_/RGO)	28.875 K	57.92	1.3143 M	0.80889	0.42651

## Data Availability

The data presented in this study are available on request from the corresponding author.

## Abbreviations

The following abbreviations are used in this manuscript:

SMO	Semiconductor metal oxide
RGO	Reduced Graphene Oxide
FESEM	Field emission scanning electron microscopy
XRD	X-ray diffractometer
QCM	Quartz Crystal Microbalance
CMUT	Capacitive Micro-machined Ultrasonic Transducer
FBAR	Film Bulk Acoustic Resonator
AC	Alternating current
EIS	Electrochemical impedance spectroscopy
RH	Relative humidity
PCB	Printed circuit board
